# Comparative Analysis of Feeding Largemouth Bass (*Micropterus salmoides*) with Trash Fish and a Compound Diet: Effects on the Growth Performance, Muscle Quality and Health Condition

**DOI:** 10.3390/ani15050654

**Published:** 2025-02-24

**Authors:** Yuanhao Yang, Yangfen Xing, Niankun Zhang, Fenggang Li, Xianfang Yan, Mingyue Zhang, Zilin Zhu, Enric Gisbert, Jishu Zhou

**Affiliations:** 1 College of Animal Science and Technology, Northwest A&F University, Yangling 712100, China; yuanhao_y@126.com (Y.Y.); 18403562599@163.com (Y.X.); 17782590167@163.com (N.Z.); 18392507099@163.com (X.Y.); zmy_1109@163.com (M.Z.); zhuzil01@163.com (Z.Z.); 2 Fisheries Research & Technology Extension Center of Shaanxi, Xi’an 710000, China; lifenggang.123@163.com; 3 Institut de Recerca i Tecnologies Agroalimentaries (IRTA), Aquaculture Program, 43540 La Ràpita, Spain; enric.gisbert@irta.cat

**Keywords:** trash fish, key performance indicators, liver and intestine condition, lipid quality nutritional index, inflammatory biomarkers

## Abstract

Two feeding strategies based on the use of trash fish (TF) and an artificial compound feed (ACF) were compared in terms of growth performance, feed efficiency, muscle quality and health status in *Micropterus salmoides*. For this purpose, fish (128 ± 14 g; n = 102) were divided into two groups and fed with the TF and ACF in triplicate for 90 days. Results showed that the growth performance and condition factor were not affected by the diet, whereas the viscerosomatic and hepatosomatic indexes in the ACF group were higher than in the TF group. The muscle from the TF group had higher levels of 20:5n-3, 22:6n-3, and total n-3 PUFA contents, which resulted in lower thrombogenicity index values. No differences in the amino acid profile were found. Regarding muscle texture properties, only the gumminess and chewiness were significantly lower in the ACF. The use of histological and gene expression biomarkers showed that fish fed TF had a healthier hepatic condition compared to the ACF. The only disadvantage of TF in the current study was the higher values of FCR in comparison to ACF.

## Simple Summary

Although the use of trash fish (TF) has been questioned for being unsustainable due to its low feed efficiency, significant water pollution, uncontrollable quality, and inconsistent nutritional composition, among other factors, TF is still used in rural areas and backyard aquaculture practices in Asia and Africa. In this study, we showed that feeding immature largemouth bass (*Micropterus salmoides*) with TF was a sound feeding strategy since it did not compromise fish growth and survival when compared to an artificial compound feed (ACF), whereas it reduced the accumulation of lipids in the liver and improved the hepatic condition, as genes associated with lipid metabolism and inflammatory response revealed. The only disadvantage of TF was the higher value of FCR in comparison to the ACF. From a consumer’s point of view, the use of TF, when compared to an ACF, did not modify the proximate composition of the muscle, whereas it improved the fatty acid profile of the muscle in terms of n-3 PUFA and reduced the thrombogenicity index. Regardless of the feeding strategy used (TF and ACF) and based on the recommendations provided by the World Health Organization, largemouth bass may be considered as a high-quality source of protein for human consumption.

## 1. Introduction

The largemouth bass (*Micropterus salmoides*), also commonly referred to as the California perch, is a fish native to California, USA. It was introduced to China in 1983 and has since become a significant species in freshwater aquaculture due to its rapid growth, high stress tolerance, palatable meat, and substantial nutritional value [1,2]. This species has also been introduced as a gamefish in more than fifty countries worldwide [3], and its farming in 2022 was reported in several countries worldwide (China, Mexico, Italy, France, Dominican Republic, Tunisia, and Algeria), even though its production in China in 2022 (805,832 t) accounted for the 99.6% of the production in the world [4].

Trash fish (TF) is commonly defined as portions of the catch that are of little or no value due to their small size or low consumer preference [5,6,7]. Trash fish primarily come from capture fisheries and are generally not consumed by humans due to their low protein content, nutritional variability, and lack of palatability. As a result, they are often discarded as by-catch, which can lead to environmental and aesthetic concerns [5,7]. The use of TF in aquaculture, used either as direct feed for carnivorous fish or as an ingredient in compound feeds, has been considered as a viable alternative for supporting fish growth [6,7,8,9]. In the initial stages of feeding largemouth bass in China and before its full domestication and adaption to compound feeds, TF was widely used as the primary feed source, and its use is still common in backyard aquaculture systems and rural areas where there is limited availability of affordable ACF. The use of trash fish as feed has led to significant improvements in the growth performance and overall health of largemouth bass [8,9,10,11]. Nevertheless, the use of trash fish as feed is generally considered as an unsustainable practice due to its low feed efficiency, significant water pollution, limited availability, uncontrollable quality, and inconsistent nutritional composition. These issues have become increasingly concerning, particularly in high-density aquaculture regions [12,13,14]. Furthermore, the breeding of largemouth bass often involves the use of large quantities of live bait or fresh frozen fish as feed. This practice not only exacerbates the overexploitation of marine resources [15] but also generates substantial organic pollution [13,16]. However, some authors have reported that no evident environmental, cost-benefit, and/or resource use advantages of commercial pellet dry feed over trash fish/low value fish as feed types [14]. Conversely, advancements in fish nutrition and the feed industry have led to significant progress in the development of ACF for largemouth bass, which are increasingly being used as alternatives to TF. Existing research has explored the nutritional requirements of largemouth bass, including their needs for protein [17,18,19,20], lipids [20], and carbohydrates [21]. These reports have focused on the development of ACF based on the nutritional requirements of largemouth bass and obtained many reliable growth and nutrition data, which show the possibility of replacing of trash fish with compounded commercial feeds in aquaculture of largemouth bass.

However, the nutritional and feed parameters of ACFs in largemouth bass are still scarce, leading to low productivity levels, especially in the middle and later production stages where problems such as slow growth, anorexia, and liver disease occur. Furthermore, anti-nutritional factors (i.e., phytates, saponins, protease inhibitors, tannins, and non-starch polisaccharides, among others) present in plant protein sources included in commercial feeds may render such diets potentially unsuitable for carnivorous species like the largemouth bass [22,23]. These dietary components may adversely affect the health and growth performance of the species, which indicated that ACFs do not always meet the species-specific nutritional requirements [12]. Several studies have reported the comparison of the two diets on the growth and health of liver and intestine and the muscle nutrition composition in largemouth bass [8,9,24,25], while more detailed information about the effect of the two of diets on largemouth black bass are essential and critical, especially on the fillet nutritional and texture quality from the consumer’s perspective, and on the evaluation whether the consumption of *M. salmoides* fed with TF meets the nutritional requirements of the consumers. Regardless of the limited use of TF in aquaculture, the comparison of both feeding strategies in terms of key performance indicators associated with growth performance, body condition, and fillet quality would provide a more comprehensive understanding of the use of TF and ACF in largemouth bass. Therefore, the aim of this experiment is to provide a theoretical basis and new data for the improvement of ACF for largemouth bass by comparing the differences in growth, muscle amino acid and fatty acid composition, muscle texture indicators, muscle, liver, and intestinal histology, and the relative expression of genes related with lipid metabolism and immunity between TF and ACF. This approach is founded on the hypothesis that feeding this carnivorous species with TF, a feeding strategy that mimics its natural dietary habits, may yield valuable nutritional insights applicable to the optimization of ACFs.

## 2. Materials and Methods

All experimental procedures in this study were strictly carried out in accordance with the guidelines of the National Council for Control of Animal Experimentation (CONCEA) for the care and use of the laboratory animals in Northwest A&F University of Animal Experimentation Ethics Committee, Yangling, China (No. DK2022007).

### 2.1. Experimental Diets

Trash fish (TF) used in the current study was the by-catch wild fish with no commercial value that were captured by artisanal fishermen from Ankang (Shaanxi province, China) during the trial (July-October). Trash fish (*Aristichthys* spp., *Hypophthalmichthys* spp.) were chopped at equal proportions into pieces of 1 cm^3^ and then stored in the refrigerator at −20 °C for later weekly use. The artificial compound feed (ACF) was a commercial compound feed with the size of 3–4 mm, purchased from Tongwei Co., Ltd. (Sichuan, China). The proximate composition (%) of the TF (fresh) and ACF (air-dried) is shown in Table 1.

### 2.2. Fish and Feeding Management

Immature largemouth bass were purchased from a commercial fish farm in AnkangCity (Shaanxi, China) and acclimated in six net cages (1 × 1 × 2 m) located in the middle of Lanhe River (Shaanxi, China) for two weeks. Then, a total of 102 healthy largemouth bass (body weight = 127.7 ± 14.4 g, mean ± standard deviation) were randomly assigned to dietary groups (AF and ACF) and cages, with three triplicates per treatment. Fish were fed by hand to apparent satiation (feeding rate: 2.5% of stocked biomass) twice a day (06:00 and 18:00 h) During the acclimation period and the nutritional trial, which lasted 90 days, water quality was regularly monitored on a daily basis (morning and evening) and water temperature, dissolved oxygen, ammonia nitrogen, and pH were kept at ca. 25.0 ± 1.0 °C, 8.8 ± 0.5 mg/L, 0.025 ± 0.005 mg/L, and 7.5 ± 0.15, respectively, thanks to the river flow (no available data on river flow during the trial). Water temperature was determined by means of a glass thermometer WNG (Changzhou Shengzhiyuan Instrument Co., Ltd., Changzhou, China), pH was measured by a pH meter PHB-5 (INESA Scientific Instrument Co., Lld., Shanghai, China), and ammonia by means of the Nessler’s ammonia test using a L3S spectrophotometer (INESA Analytical Instrument Co., Ltd., Shanghai, China). Dissolved oxygen levels were determined following the idometric method as described in ISO 5813:1983. The photoperiod followed natural changes according to the season (July to October at 32.484814 N–108.88303 E).

### 2.3. Fish Sampling, and Color Measurement of the Skin and Muscle

At the end of the 90-day experimental period, fish were fasted for 24 h before sampling, and all fish were individually measured for body weight (BW, g) and total length (cm) after their anesthesia with eugenol (dose: 0.01 g/L) (Yisenyuan Plant Spices Co., Ltd., Jiangxi, China). After body individual morphometrics, the skin color from the dorsal, lateral, and ventral part of the fish was measured by a 3 NH Colorimeter (NS800, Sanenshi Technology Co., Ltd., Shenzhen, China). Then, fish were euthanized with an overdose of anesthetic, skinned, and the color of the dorsal muscle was also determined by the same procedure. In all cases, the brightness, redness, and yellowness of the skin and fillet were determined by the L*, a*, and b* parameters, respectively. After that, fish were dissected on an ice-cold dissecting tray, and the weight of the viscera, liver, and intraperitoneal fat was measured. Then, the dorsal muscles were dissected (2 cm^2^) and the muscle texture, pH, and water-holding capacity of the muscles of three fish from each net cage were analyzed within 48 h. Furthermore, the dorsal muscles from another three fish from each net cage were stored at −80 °C for analysis of proximate composition and amino acid and fatty acid composition. The muscle, liver, and middle intestine of three fish from each net cage were also sampled and fixed in the 4% formaldehyde solution for histological observation, while a piece of liver (ca. 300 mg) was also dissected and immediately stored at −80 °C for qPCR analyses.

### 2.4. Growth, Feed Efficinency, and Biological Parameters

The following formulae were used to evaluate different key performance indicators associated with somatic growth, body condition, and feed efficiency:

Weight gain rate (WGR, %) = 100 × (final BW − initial BW)/initial BW.

Feed conversion rate (FCR) = food intake/(final biomass − initial biomass).

Survival rate (SR, %) = 100 × number of final fish/number of initial fish.

Fulton’s condition factor (K, g/cm^3^) = 100 × fish BW/body length^3^.

Viscerosomatic index (VSI, %) = 100 × visceral weight/BW.

Hepatosomatic index (HSI, %) = 100 × liver weight/BW.

Intraperitoneal fat body index (IPFI, %) = 100 × hepatosomatic body weight/BW.

The meat content (MC, %) of each fish was measured according to the method of the national standard GB/T18654.9-2008 (Ministry of Agriculture, Pekin, People’s Republic of China), which considers the fillet yield (g) with regards to the body weight of fish (g).

### 2.5. Proximate Composition, Amino Acid and Fatty Acid Profiles

The proximate composition of AF, ACF, and muscle samples was analyzed following the methodology proposed by the Association of Official Analytical Chemists [26]. In brief, the moisture content was determined by drying the sample at 105 °C, the crude protein was determined by the Kjeldahl method, the crude lipid was determined by the ether extract Soxtec system, and the crude ash was measured by burning at 550 °C in a muffle furnace.

The amino acid (AA) composition of the diets (Table 2) and muscular tissue of the fish were determined by the Sichuan Weil Testing Technology Co., Ltd. (Chengdu, China) with the Symkam amino acid automatic analyzer (Seccam, Germany) according to standard procedures GB/T 18246-2019 and GB/T 5009.124-2003, respectively (Ministry of Agriculture, People’s Republic of China). From a consumers’ nutritional point of view, the muscle protein quality of largemouth bass fed both diets was assessed using AA scoring patterns for evaluating proteins proposed by the FAO/WHO Expert Committee [27]. In particular, the following AA indexes were used for the evaluation of the nutritional quality of the muscle protein: Amino acid mass ratio (mg/g N) = AA content (fresh sample)/crude protein content (fresh sample) × 6.25 × 1000; the essential amino acid index, EAAI) = (Lys t/Lys s × 100 × Leu t/Leu s × 100 × … Val t/Val s × 100) ^1/n^, where t represents the amino acid (mg/g) of the test protein, and s represents the egg protein amino acid (mg/g), n represents the number of amino acids compared; and the ratio of branched-chain AA to the aromatic AA (BAA/AAA) [28].

Lipids were extracted from samples (diets and tissues) with a solution of chloroform-methanol (volume ratio 2:1) and were esterified in KOH-methanol solution (0.4 mol/L) for 30 min, while 2 mL of the deionized water was added in the mixture solution, followed by the centrifugation of 3000 rpm for 5 min. After centrifugation, the upper layer (1 μL) was injected into a Gas Chromatograph system (Agilent 7820a, Agilent Technologies, Santa Clara, CA, USA) to determine the fatty acid composition using a two-stage thermal gradient from 50 °C (injection temperature) to 150 °C after ramping at 40 °C/min and holding at 250 °C after ramping at 2 °C/min. Helium (1.2 mL/min) was used as the carrier gas and on column injection and flame ionization detection at 250 °C. Each fatty acid was compared with a known standard (47015-U, Sigma-Aldrich, Inc., St. Louis, MI, USA), and the fatty acid content was calculated by the area normalization method as a percentage of total fatty acid [29]. The nutritional quality of the lipid in muscle was analyzed using the following formulae [30]:

Index of atherogenicity (AI) = [(C12:0 + (4 × C14:0) + C16:0)]/(ƩMUFA + Ʃn-6 PUFA + Ʃn-3 PUFA).

Index of thrombogenicity (TI) = (C14:0 + C16:0 + C18:0)/[(0.5 × ƩMUFA) + (0.5 × Ʃn-6 PUFA) + (3 × Ʃn-3 PUFA) + (Ʃn-3 PUFA/Ʃn-6 PUFA)].

Hypocholesterolemic/hypercholesterolemic fatty acids ratio (h/H) = (C18:1n-9 + C18:3n-6 + C18:3n-3 + C20:5n-3 + C22:6n-3)/(C12:0 + C14:0 + C16:0).

Health promotion index (HPI, %) = (ƩMUFA + ƩPUFA)/(C12:0 + 4 × C14:0 + C16:0).

**Table 2:** The amino acids (AA) composition of trash fish (TF) and artificial compound feed (ACF) expressed as percentage (%) on a dry weight basis used in largemouth bass (*Micropterus salmoides*).

EAA	TF	ACF	LBrq ***	NEAA	TF	ACF
Valine	3.59	2.49	2.43	Aspartate	7.26	4.77
Methionine	2.15	1.27	1.22	Serine	3.09	1.97
Isoleucine	3.18	2.09	2.30	Glutamic acid	11.61	7.33
Leucine	5.52	3.76	2.52	Glycine	6.59	3.79
Phenylalanine	3.05	2.12	2.02	Alanine	5.07	3.44
Lysine	6.28	4.22	3.07	Cystine	0.54	0.61
Histidine	2.11	1.48	1.26	Proline	4.30	2.75
Arginine	4.57	2.94	1.91	Tyrosine	2.56	1.54
Threonine	3.32	2.46	1.43			
ΣEAA *	33.77	22.83	18.16	ΣNEAA **	41.03	26.20

Abbreviations: * ΣEAA means the total essential amino acids. ** ΣNEAA means the total non-essential amino acids. *** LBrq stands for the nutritional requirements in essential amino acids in largemouth bass [31,32].

### 2.6. Analysis of the Muscle Texture Profiles

Texture parameters of the muscular tissue, like the hardness, adhesiveness, cohesiveness, springiness, gumminess, and chewiness, in largemouth bass feed experimental diets were determined by means of TMS-Pilot Precision texture (Food Technology Corporation, Sterling, VA, USA). In brief, texture parameters were analyzed with the probe P5, using a pre-test rate of 3 mm/s, two consecutive tests at a test rate of 1 mm/s, a compression degree of 50%, and a residence interval 5 s. All texture analyses were run at an ambient temperature of 18–20 °C. The shear force of the muscle was determined using an initial force of 0.1 N (return speed were 60 mm/min), the contact induction was 5 gf, and the testing speed was 1 mm/s. The water-holding capacity and the pH of the muscle were determined by the method of GB/T5009.3-2003 (Ministry of Agriculture, Pekin, People’s Republic of China) and Du et al., respectively [33].

### 2.7. Histological Analyses

The samples of liver, intestine, and muscle tissue fixed in solution were taken out and washed with clean water; then they endured dehydration in different alcohol solutions and were then embedded in the paraffin. Tissue sections were performed, and sample tissue sections were 4 µm thick. Hematoxylin-eosin (HE) staining was used to observe the stained sections. After imaging, Image-Pro Plus 6.0 software (Media Cybemetics, Rockville, MD, USA) was used to measure the intestinal villus length, villus width, and muscular layer thickness in each section.

### 2.8. Quantitative Real Time PCR

Total RNA extraction and the reverse transcription of the RNA were performed using standard protocols. The CFX96 Real-Time Quantitative PCR Detection System (Bio-Rad Laboratories, Hercules, CA, USA) was used for gene expression real-time quantitative PCR analyses. In brief, the 20 μL reaction system contained 0.6 μL primers, 1 μL diluted cDNA, 10 μL 2 × SYBR^®^ Premix Ex TaqTM II, and 7.8 μL sterile non-enzymatic water. The qPCR reaction conditions were as follows: an initial activation step at 95 °C for 30 s, followed by 40 cycles (95 °C for 15 s, 60 °C for 15 s). The CT values of target and housekeeping genes were obtained using amplification curves after PCR reactions, and the relative expression of the target gene in each group was calculated according to the formula 2^−△△CT^ [34]. The following genes and associated physiological process were selected in the current study: lipid metabolism (*ppar-α*, *cpt1*), lipid catabolism (*ppra-γ*, *fas*); anti-inflammatory cytokines (*il-10, tfg-β1*); and pro-inflammatory cytokines (*il-1β*, *tnf-α*). *β-actin* was used as house-keeping gene. The PCR primer sequences of each gene are shown in Table 3.

### 2.9. Statisticial Analyses

Data are presented as mean ± standard error of the mean (SEM). After checking their normality (Kolmogorov–Smirnov test) and the homogeneity of variances (Levene’s test), data from the TF and ACF groups were compared using a *t*-test and the statistical significance was determined at *p* < 0.05. According to data variability reported in similar studies comparing TF and ACF [5,6,8], the minimum sample size, needed for finding statistical differences between groups and excluding the influence of random errors, was determined based on Type 1 α risk, *p* = 0.05, and Type 2 β risk, equal to 20% (the statistical power {1 β} = 80%). All statistical procedures were run using SPSS software version 22.0 for Windows (IBM Corp., Armonk, NY, USA), whereas graphics were prepared by means of GraphPad Prism Software version 7.0 (GraphPad Software, Boston, MA, USA).

## 3. Results

### 3.1. Growth, Feed Efficiency and Body Condition Parameters in Largemouth Bass

Table 4 shows the results in terms of key performance indicators related to growth performance, feed efficiency, survival, and body condition somatic indexes in immature largemouth bass during their on-growing period fed experimental diets. Regarding somatic growth, regardless of the feed used, TF vs. ACF, there were no statistically significant differences in terms of BWf, WGR, and K between groups (*p* > 0.05). Compared to the TF group, FCR values were lower in largemouth bass fed the ACF (*p* < 0.05). Regarding body condition indexes, VSI and HSI were significantly higher in fish fed the ACF compared to their congeners fed the TF (*p* < 0.05), whereas no differences were found in terms of IFBI and MC between both groups (*p* > 0.05). Regardless of the diet administered, there were not statistically significant differences in terms of survival rates (*p* > 0.05).

### 3.2. Muscle Proximate Composition, Amino Acid and Fatty Acid Profiles

As shown in Table 5, the use of TF and ACF did not affect the proximate composition of the muscle in largemouth bass during their on-growing period, as there were no significant differences in the crude lipid, crude protein, moisture, and ash levels between the two groups (*p* > 0.05). Furthermore, the AA composition of the muscle was stable regardless of the diet administered since no statistically significant differences were found in the content of EAA nor in the levels of NEAA (Table 6; *p* > 0.05).

The evaluation of the protein quality by means of the AA profile scoring proposed by the FAO/WHO of the muscle from largemouth bass fed TF and ACF is shown in Table 7. In particular, the EAA muscle content of immature fish fed both diets was higher than the recommended values by the FAO/WHO, whereas when comparing current data with egg protein, only the levels of threonine and lysine were higher than those of the egg protein. The EAAI and BAA/AAA were not significantly different among the two groups (*p* > 0.05). Furthermore, the nutritional quality of the muscle in largemouth bass was very high, as the EAA scores in both groups were higher than 1, and the AA chemical score for each EAA was also higher than 0.6. These results were also supported by the chemical score values of EAA, especially those for lysine, which were higher than 1. The nutritional quality of the protein in largemouth bass fed TF and ACF did not vary significantly (Table 7; *p* > 0.05).

Regarding the fatty acid composition of the muscle of largemouth bass fed both diets, the contents of 18:0, 20:4n-6, 20:5n-3, and 22:6n-3 in the muscle of fish fed the ACF were significantly lower compared with the TF group (Table 8; *p* < 0.05). Similarly, the levels of 14:0, 16:1n-7, 18:1n-9, 18:2n-6, and 20:2n-6 in muscle of fish from the ACF group were higher in comparison to the TF group, whereas the ratio of n-3/n-6 significantly decreased in the muscle of largemouth bass fed the ACF in comparison to their congeners fed TF (*p* < 0.05). Regarding the lipid nutritional quality indexes, only the TI in the ACF group was significantly higher than in the TF group (*p* < 0.05), whereas the rest of indexes (AI, HH, and HPI) did not vary between the two groups (*p* > 0.05).

### 3.3. Skin and Muscle Colour Features

As shown in Table 9, there were no significant differences in the brightness (L*), redness (a*), and yellowness (b*) values of different body regions of the skin (dorsal, lateral, and ventral areas), nor in the muscle between the two dietary groups (*p* > 0.05).

### 3.4. Muscle Textural Properties

Muscle gumminess and chewiness in largemouth bass from the ACF group were significantly lower than in the TF group (Table 10; *p* < 0.05). There were no significant differences in muscle hardness, adhesiveness, cohesiveness, springiness, shear force, water-holding capacity, and pH between the two groups (Table 10; *p* > 0.05).

### 3.5. Histological Organization of Target Tissues (Muscle, Liver and Intestine)

**Figure 1:**
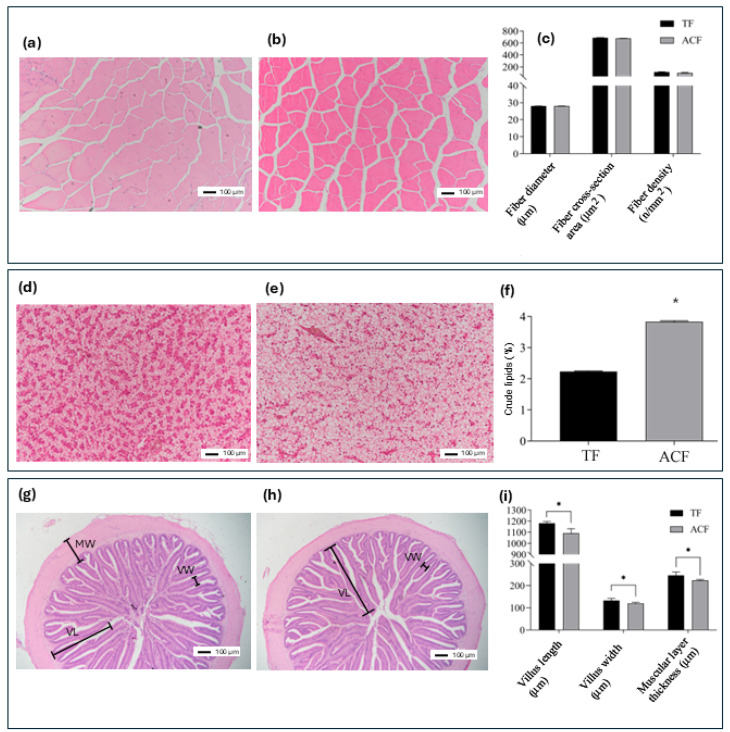
Histological organization of the muscle (**a**,**b**), liver (**d**,**e**), and intestine (**g**,**h**). The histology observation of the muscle, liver, and intestine by HE staining and the calculation of the characteristic parameters of largemouth bass (*Micropterus salmoides*) fed trash fish (AF) and an artificial compound diet (ACF) (n = 6 per dietary group; 3 per cage). Muscle fiber characteristics in terms of fiber diameter, fiber-cross sectional area, and fiber density (mean ± SEM) are also presented (**c**). Levels of crude lipids (mean ± SEM) in liver samples are included for supporting histological results (**f**). Morphometric features of the intestine (mean ± SEM) in terms of villi height and length, and width of the muscular layer surrounding the intestine (**i**). The asterisk denotes statistically significant differences between both groups (*t*-test, *p* < 0.05). Staining: hematoxilyn-eosin.

The skeletal muscle in largemouth bass fed ACF and TF was similar between both groups, with muscular fibers organized in well-defined muscular bands with a high degree of evenness and regularity (Figure 1a,b). There were no significant differences in muscle fiber diameter, fiber cross-sectional area, and muscle fiber density between the TF and ACF groups (*p* > 0.05; Figure 1c).

The histological organization of the hepatic parenchyma in largemouth bass fed TF and ACF was similar. Hepatocytes were organized in tightly packed, interconnected plates surrounding the veins, and the hepatic parenchyma was enclosed by a thin layer of fibroconnective tissue. No melanomacrophage centers or lymphocyte infiltrations were found, which indicated a healthy hepatic condition in both dietary treatments. However, the only difference between the groups was the level of lipid accumulation within hepatocytes, which was larger in fish fed ACF than in those animals from the TF group (Figure 1d,e). These results were supported by higher levels of crude lipids in the liver (Figure 1f; *p* < 0.05).

As depicted in Figure 1g,h, in both experimental groups, the intestine showed a normal histological organization. In particular, the intestinal folds were covered with a simple columnar epithelium characterized by basal nuclei, basophilic cytoplasm, and distinct microvilli in both dietary groups. Goblet cells, abundant among the enterocytes, actively produced and secreted substantial amounts of mucus into the intestinal lumen. There were no signs of inflammatory disorders or enteritis associated with any of the experimental diets. The lamina propria, submucosa, and tunica muscularis exhibited normal structural organization, and no lipid deposits were observed within the enterocytes or the vascular system. The morphometric analysis of the intestinal villi revealed that the length and width of villi, as well as the thickness of the intestinal muscular layer, were lower in fish fed ACG compared to those specimens fed TF (Figure 1i; *p* < 0.05).

### 3.6. Expression of Hepatic Lipid Metabolism and Inflammatory Cytokine Genes

**Figure 2:**
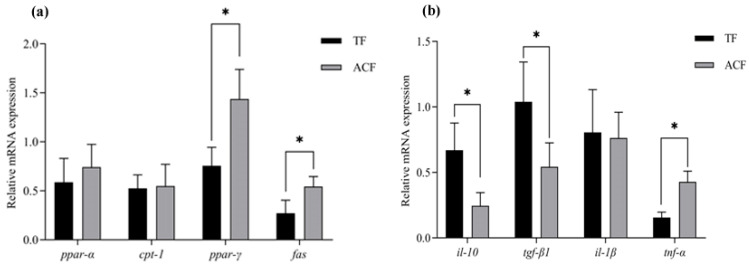
Expression levels (mean ± SEM) of genes related with lipid metabolism (**a**) and inflammatory cytokine (**b**) in the liver of largemouth bass (*Micropterus salmoides*) fed trash fish (AF) and an artificial compound diet (ACF) n = 6 per dietary group; 3 per cage). The asterisk denotes statistically significant differences between both groups (*t*-test, *p* < 0.05).

The relative expression of selected genes involved in lipid catabolism (*ppar-α* and *cpt-1*) did not differ between the groups (Figure 2a; *p* > 0.05). In contrast, the relative expression of *ppar-γ* and *fas* genes associated with lipid anabolism was significantly up-regulated in the liver of fish fed the ACF compared to the TF group (Figure 2a; *p* < 0.05). Regarding inflammatory cytokines, the gene expression of selected anti-inflammatory cytokins like *il-10* and *tgf-β1* were significantly down-regulated in the ACF group compared with the TF group (Figure 2b; *p* < 0.05), whereas the expression of *tnf-α*, a proinflammatory cytokine, was significantly up-regulated in the liver of fish fed the ACF. In contrast, no significant differences were found in the expression levels of *il-1β* between both groups (Figure 2b; *p* < 0.05).

## 4. Discussion

Low-value or trash fish is commonly used as feed utilized in cages and backyard aquaculture practices across Asia and Africa [6]. However, its use is debated due to concerns about the sustainability of these farming practices and their associated environmental impacts [6,14]. In this study, we evaluated the efficacy of using an ACF compared to TF in largemouth bass during their on-growing period in terms of fish performance and condition and provided insight into the effectiveness of current feeding practices.

Regardless of environmental and management factors, one of the main factors affecting fish growth performance is the nutrient composition of feeds. Under current experimental conditions, we showed that the WGR of farmed largemouth bass in cages and fed TF and ACF was similar, which were consistent with previous results found in several marine and freshwater species like the large yellow croaker (*Larimichthys crocea*) [12], largemouth bass [8,11,12], leopard mandarin fish (*Siniperca scherzeri*) [39], Asian seabass (*Lates calcarifer*), and tiger grouper (*Epinephelus fuscoguttatus*) [14]. However, these results differed from those reported by Mai et al. [40] comparing TF (mud carp, *Cirrhinus molitorella*) and a commercial ACF in which largemouth bass fed TF grew better than those fed the ACF, results that were attributed to the better nutritional value of TF compared to the ACF. Regarding feed efficiency parameters, FCR values were lower in the ACF group compared to their congeners fed the TF, results that are mainly due to the higher moisture levels of TF compared to the ACF (77.7 vs. 7.8%). In this context, to achieve a similar BWf, fish fed with TF must consume larger amounts of food compared to those in the ACF group, which accounts for the higher feed conversion ratio (FCR) values observed. These results are in line with previous trials comparing these two types of feeding practices [41,42,43].

From a consumer point of view, the contents of crude protein and lipids, as well as the AA and fatty acid profiles of fish, are of major importance [27,39,44], being easily influenced by the nutritional value of feeds. In this trial, farming largemouth bass in cages using TF or ACF did not vary the proximate composition of the fillet, which agreed with previous results in other aquatic species like large yellow croaker [45] and snakehead (*Channa argus)* [46]. The AA composition of the fillet is also a good and reliable indicator of the nutritional quality of fish [27]. Under current experimental conditions and similarly to other studies [40,41], there were no differences in the EAA profile in largemouth bass fed TF and ACF, values equivalent to or even slightly higher than the recommendations provided by the World Health Organization [27], which highlights the high protein quality of the muscle in this carnivorous freshwater species.

When considering the fat content of fish fillets, the fatty acid composition of the fish muscle is more significant for consumer health than the overall lipid levels. This is because fatty acids can have either beneficial or detrimental effects on the prevention and treatment of diseases [47,48]. In this context, fish serve as the primary source of n-3 HUFA, particularly EPA and DHA, which are essential for human health [8,27,49]. Consist with previous results in largemouth bass [8,50], large yellow croaker [9], and hybrid grouper (*Epinephelus fuscoguttatus* × *Epinephelus lanceolatus*) [51], the current study showed lower EPA and DHA contents in the muscle of immature largemouth bass fed the ACF compared to their congeners fed the TF, which might be due to the lower contents in fish oil in the ACF used in this study. This hypothesis is supported by the fact that the body fatty acid composition in fish generally mirrors that of the feed [48,52]. This is of special relevance since regardless of the questionable use of TF in aquaculture practices, its use may have a beneficial health effect for local communities using this feeding approach, as indicated by data on the levels of n-3 HUFA and lipid nutritional quality indexes like the index of thrombogenicity. Furthermore, under current experimental conditions, the fillet fatty acid profile (SFA, MUFA, n-3 PUFA, and n-6 PUFA) of largemouth bass fed TF and ACF resembled that found in other studies when this species was fed with ACF rich in fish oil, whereas they differed from those values in fish fed diets based on corn and sunflower oils. In particular, largemouth bass fed ACF rich in corn and sunflower oils showed lower levels of SFA, n-3 PUFA, and n-6 PUFA and a higher content of MUFA compared to the current study [53]. These values found in largemouth bass are slightly higher than those reported in farmed carnivorous species like striped bass (*Morone saxatilis*), channel catfish (*Ictalurus punctatus*), white sturgeon (*Acipenser transmontanus*), or green sturgeon (*Acipenser medirostris*) [54]. When compared to other carnivorous species, the fillets of largemouth bass fed TF and ACF showed lower contents of SFA and n-3 PUFA than Northern pike (*Esox lucius*), as well as higher levels of MUFA and n-3 PUFA [55]. The above-mentioned differences between different fish species may be attributed to their trophic level and diet [53,54,55,56].

From a consumer’s point of view, the visual appeal of fish plays a crucial role in its marketability, often outweighing considerations of its nutritional value. In this context, the colour of the skin and muscle in the fish are based on dietary pigments [57,58]. In the current study, the colour of different skin body regions remained unaltered regardless of the tested diet, results that were similar to those found in the muscle colour [12,50]. These results are of relevance since there is a close relationship between skin and muscle pigmentation and diet, as has been documented in several farmed species [57,58,59,60], which indicates that the supplementation of commercial feeds with pigments is not needed in terms of supporting skin and muscle colour quality properties. Under the scope of consumer perception of aquaculture products, the texture of the muscle is a primary determinant of consumer satisfaction. Different diets can affect muscle structure, firmness, and chewiness, which are important for sensory acceptance [60,61]. In particular, the hardness, adhesiveness, cohesiveness, and springiness of the muscle are indicators of muscle texture quality [61,62], whereas the higher values of chewing and the gumminess are indicative of good textural quality [63,64]. Present results showed lower muscle gumminess and chewiness values in the muscle of largemouth bass from the ACF group compared with the TF group, which may indicate that fish fed with TF have a better muscle quality than their counterparts fed with ACF. This is in line with previous results in hybrid grouper and largemouth bass [51,65]. Furthermore, the texture properties of the muscle, and especially its tenderness, water-holding capacity, and texture, depend on the muscle fibres [66]. In this sense, muscle hardness is negatively correlated with muscle fibre diameter and positively correlated with muscle fibre density [67], whereas the smaller diameter of muscle fibres and higher fibre density are linked to higher muscle hardness and better taste [68]. When comparing the results of muscle texture properties coupled with the histological data on diameter and density of muscle fibres, we showed that the use of TF or ACF did not affect fillet quality, which is of relevance from the consumer’s perspective.

When testing a diet, its evaluation from a consumer point of view should be coupled with its assessment in terms of fish condition. In the present study, both dietary strategies were also compared by means of the assessment of histological features from digestive organs like the liver and intestine, since these two digestive organs respond rapidly and sensitively to nutritional disorders [69,70]. Regarding the intestine, size of villi in terms of length and width, as well as the width of the *tunica muscularis*, was lower in fish fed the ACF in comparison to the TF group. Similar results were found in snakehead (*Channa argus*) and marbled flounder (*Pseudopleuronectes yokohamae*) [46,59]. Such differences between TF and ACF in terms of histological features of the intestine may be attributed to the different texture and digestible properties between diets. In particular, TF may be more complex to digest than the ACF due to their content in indigestible elements like bones, spines, or skin, which would require a thicker gut wall and larger villi for guaranteeing the absorption of nutrients [71,72,73]. Regardless of these differences in villi size, no differences in somatic growth were reported between both groups; this may be due to the feeding strategy employed, which supported proper growth regardless of the feed used. Concerning the liver, largemouth bass fed ACF showed higher HSI values than fish fed TF, results that were correlated with a higher accumulation of lipid inclusions in the hepatic parenchyma and larger crude lipid contents. These findings may be attributed to the different content in crude lipids between TF and ACF (3.9 vs. 12.1%, respectively) [9,51,73] and lipid composition [9]. Fat accumulation in the liver depends on the incorporation of exogenous fatty acids and their de novo lipogenesis. In this context, the higher accumulation of lipids in the liver of fish fed ACF were supported by changes in selected genes associated with lipid metabolism like *ppar-γ* and *fas*. For instance, *ppar-γ* is involved in adipogenesis and the accumulation of fat in the liver [74,75], whereas *fas* regulates *de novo* lipogenesis and adipocyte differentiation [76,77,78]. The increase in the above-mentioned genes may explain the higher accumulation of fat deposits in the liver, whereas other genes involved in fatty acid oxidation and lipid catabolism, like *ppar-α* and *cpt-1*, remained stable between both dietary groups [78]. The use of TF compared to ACF was also evaluated in terms of the modulation of genes involved in inflammatory responses, which are proxy biomarkers of health condition and the host’s immune response [79,80]. Under current experimental conditions and in the line with the results of the expression of hepatic genes involved in lipid metabolism, we found a down-regulation of anti-inflammatory cytokines (*il-10* and *tgf-β1*) coupled with the up-regulation of the pro-inflammatory cytokine of *tnf-α*, which showed a healthier hepatic condition in fish fed TF compared to ACF [81]. Thus, the combination of histological and gene expression results indicated that use of TF may be a healthier feeding strategy with regards to the liver condition, especially due to their lower lipid content, whereas the potential long-term use of the tested ACF on the hepatic condition and fish health needs to be further investigated.

## 5. Conclusions

Although the use of TF compared to ACF has been questioned for being unsustainable due to its low feed efficiency, significant water pollution, uncontrollable quality, and inconsistent nutritional composition, among other factors, this feeding practice based on the use of TF is still used in rural areas and backyard aquaculture practices in Asia and Africa. In the present study, we showed that feeding largemouth bass with TF (*Aristichthys* spp., *Hypophthalmichthys* spp.) was a sound feeding strategy since it did not compromise fish growth and survival, whereas it reduced the amount of lipid accumulation in the liver and improved its condition, as gene markers associated with lipid metabolism and inflammatory response revealed. The only disadvantage of TF in the current study was the higher values of FCR in comparison to ACF. Given that TF are considered as a by-catch with no economic value for human consumption, their use as a feeding strategy will be feasible in terms of feeding costs when compared to ACF, which must be purchased by local fish farmers, although the environmental footprint of this practice in terms of water quality, phosphorous dejections, and disease outbreaks need to be carefully evaluated in further studies. Furthermore, considering that TF are widely used in rural areas, the results of the present study cannot be directly extrapolated to all the regions in which this practice is conducted since the nutritional value of TF may vary depending on the season of capture, the by-catch species considered, and the size and/or stage of maturity of TF among other factors. Thus, the comparison of this feeding practice with commercial ACF should be also set up based on the TF used and the local ACF available. From a consumer’s point of view, the use of TF when compared to an ACF did not modify the proximate composition of the muscle, whereas it improved the fatty acid profile of the muscle in terms of n-3 PUFA, DHA, and EPA and reduced the thrombogenicity index. Regardless of the feeding strategy used (TF and ACF) and based on the recommendations provided by the World Health Organization, largemouth bass may be considered as a high-quality source of protein for human consumption.

## Figures and Tables

**Table 1:** The proximate composition (%, mean ± SEM) of the trash fish (TF, fresh) and the artificial formulated feed (ACF, dry weight) used as feed for largemouth black bass (*Micropterus salmoides*).

Diet	Crude Protein	Crude Lipid	Moisture	Ash
Trash fish	16.41 ± 0.85	3.88 ± 0.02	77.69 ± 1.01	1.02 ± 0.01
ACF	53.20 ± 0.11	12.05 ± 0.08	7.67 ± 0.10	7.56 ± 0.04

**Table 3:** Primer sequences for real-time qPCR analysis used for gene expression of lipid metabolism, anti-inflammatory and pro-inflammatory cytokines in largemouth bass (*Micropterus salmoides*) fed experimental feeds.

Gene	Forward Primer (5→3)	Reverse Primer (5→3)	Size (bp)	E (%)	Refs.
*ppar-α*	CCACCGCAATGGTCGATATG	TGCTGTTGATGGACTGGGAAA	144	104.3	[35]
*cpt-1*	CATGGAAAGCCAGCCTTTAG	GAGCACCAGACACGCTAACA	128	98.8	[35]
*ppar-γ*	AGCAGACATCCGCCCTAA	ACCTCGATCACGCCGTAC	160	99.7	[36]
*fas*	TGATGATAACTGGCTTCGG	TCAAACCTGGACCCTACCT	86	100.1	[36]
*il-10*	CGGCACAGAAATCCCAGAGC	CAGCAGGCTCACAAAATAAACATCT	119	113.6	[37]
*tgf-β1*	GCTCAAAGAGAGCGAGGATG	TCCTCTACCATTCGCAATCC	118	95.6	[37]
*il-1β*	CGTGACTGACAGCAAAAAGAGG	GATGCCCAGAGCCACAGTTC	166	101.3	[37]
*tnf-α*	CTTCGTCTACAGCCAGGCATCG	TTTGGCACACCGACCTCACC	161	105.7	[37]
*β-actin*	AAAGGGAAATCGTGCGTGAC	AAGGAAGGCTGGAAGAGGG	184	100.1	[38]

**Table 4:** Key performance indicators (KPIs) linked to somatic growth performance, feed efficiency, body condition, and survival (mean ± SEM) of largemouth bass (*Micropterus salmoides*) fed trash fish (AF) and an artificial compound diet (ACF). The asterisk denotes statistically significant differences between both groups (*t*-test, *p* < 0.05).

KPI	TF	ACF	KPI	TF	ACF
BWi (g)	136.20 ± 16.10	119.20 ± 6.20	K	2.57 ± 0.27	2.49 ± 0.09
BWf (g)	366.70 ± 57.80	317.50 ± 18.70	VSI (%)	7.69 ± 0.65	8.67 ± 0.70 *
WGR (%)	168.23 ± 12.87	166.87 ± 19.50	HSI (%)	1.50 ± 0.12	2.34 ± 0.13 *
FCR	2.98 ± 0.14	1.44 ± 0.03 *	IFBI (%)	2.05 ± 0.09	2.25 ± 0.31
SR (%)	85.95 ± 3.83	90.20 ± 12.25	MC (%)	76.27 ± 1.24	75.44 ± 0.95

Abbreviations: BWi and BWf, initial and final body weight; WGR, weight gain rate; FCR, feed conversion ratio; SR, survival rate; K, Fulton’s condition factor; VSI, viscerosomatic index; HIS, hepatosomatic index; IFBI, intraperitoneal body fat index; MC, body meat content.

**Table 5:** Proximate composition (%, mean ± SEM; n = 9 per dietary group; 3 per cage) of the muscle of largemouth bass (*Micropterus salmoides*) fed trash fish (AF) and an artificial compound diet (ACF).

Experimental Group	Moisture	Crude Protein	Crude Lipid	Ash
TF	77.07 ± 0.46	19.14 ± 0.04	1.08 ± 0.06	1.29 ± 0.01
ACF	76.92 ± 0.20	19.39 ± 0.06	1.32 ± 0.05	1.28 ± 0.01

**Table 6:** Amino acid profile (%, mean ± SEM; n = 9 per dietary group; 3 per cage) of the muscle of largemouth bass (*Micropterus salmoides*) fed trash fish (AF) and an artificial compound diet (ACF).

EAA	TF	ACF	NEAA	TF	ACF
Threonine	0.91 ± 0.01	0.91 ± 0.02	Aspartate	2.12 ± 0.01	2.10 ± 0.04
Valine	0.94 ± 0.02	0.95 ± 0.05	Serine	0.80 ± 0.02	0.79 ± 0.02
Methionine	0.62 ± 0.02	0.62 ± 0.01	Glutamic	3.13 ± 0.04	3.14 ± 0.05
Isoleucine	0.88 ± 0.02	0.91 ± 0.03	Glycine	1.08 ± 0.04	1.02 ± 0.04
Leucine	1.63 ± 0.02	1.64 ± 0.03	Alanine	1.15 ± 0.13	1.22 ± 0.02
Phenylalanine	0.84 ± 0.02	0.87 ± 0.03	Cystine	0.11 ± 0.01	0.11 ± 0.01
Lysine	1.85 ± 0.07	1.91 ± 0.04	Tyrosine	0.73 ± 0.02	0.74 ± 0.02
Histidine	0.49 ± 0.03	0.52 ± 0.02	Proline	0.67 ± 0.08	0.66 ± 0.04
Arginine	1.20 ± 0.01	1.17 ± 0.03			
ƩEAA	9.35 ± 0.15	9.51 ± 0.26	ƩEAA/ƩAA	95.61 ± 0.53	97.23 ± 0.82
ƩNEAA	9.78 ± 0.21	9.78 ± 0.20	ƩEAA/ƩNEAA	47.29 ± 0.13	47.14 ± 0.36

Abbreviations: EAA, essential amino acids; NEAA, non-essential amino acids.

**Table 7:** Comparison of the protein quality (mean ± SEM) based on AA scores proposed by the WHO/FAO and egg protein content of the muscle in largemouth bass (*Micropterus salmoides*) fed trash fish (AF) and an artificial compound diet (ACF).

AA	AA Mass Ratio (mg/g N)				EAA Score		EAA Chemical Score	
	TF	ACF	WHO Score (mg/g N)	Egg Protein (mg/g N)	TF	ACF	TF	ACF
Thr	292.1 ± 4.6	294.6 ± 7.9	250	292	1.17 ± 0.02	1.18 ± 0.03	1.00 ± 0.02	1.01 ± 0.03
Val	313.0 ± 9.7	316.7 ± 15.5	310	411	1.01 ± 0.03	1.02 ± 0.05	0.74 ± 0.02	0.75 ± 0.04
Iso	283.0 ± 9.9	294.7 ± 10.9	250	311	1.13 ± 0.04	1.18 ± 0.04	0.86 ± 0.03	0.89 ± 0.03
Leu	524.4 ± 9.5	529.4 ± 12.2	440	534	1.19 ± 0.02	1.20 ± 0.03	0.98 ± 0.02	0.99 ± 0.02
Lys	595.4 ± 20.4	615.4 ± 16.5	340	411	1.75 ± 0.06	1.81 ± 0.05	1.35 ± 0.05	1.40 ± 0.04
Met + Cys	234.7 ± 6.3	235.6 ± 3.3	220	386	1.07 ± 0.03	1.07 ± 0.01	0.61 ± 0.02	0.61 ±0.01
Phe + Tyr	507.3 ± 9.7	518.3 ± 13.4	380	565	1.34 ± 0.03	1.36 ± 0.04	0.90 ± 0.02	0.92 ± 0.02
Total	2739 ± 51	2794 ± 73	2190	2960				
EAAI	90.19 ± 1.71	91.86 ± 2.44						
BAA/AAA	2.19 ± 0.04	2.18 ± 0.04						

Abbreviations: EAAI, essential amino acid index; BAA/AAA, ratio of branched-chain amino acids to aromatic amino acids.

**Table 8:** Fatty acid composition (%, mean ± SEM) and lipid nutritional quality indexes (mean ± SEM; n = 9 per dietary group; 3 per cage) of the muscle of largemouth bass (*Micropterus salmoides*) fed trash fish (AF) and an artificial compound diet (ACF). The asterisk denotes statistically significant differences between both groups (*t*-test, *p* < 0.05).

Fatty Acids	TF	ACF
14:0	1.50 ± 0.17	1.81 ± 0.14 *
16:0	26.14 ± 0.59	25.24 ± 2.15
18:0	0.93 ± 0.21	0.35 ± 0.08 *
SFA	28.56 ± 0.90	27.39 ± 2.30
16:1n-7	3.20 ± 0.29	4.75 ± 0.33 *
18:1n-9	12.20 ± 1.35	18.22 ± 7.16 *
MUFA	15.40 ± 1.59	22.98 ± 6.88 *
18:2n-6 (LA)	19.82 ± 1.93	26.28 ± 2.96 *
20:2n-6	0.76 ± 0.09	1.43 ± 0.22 *
20:4n-6 (ARA)	6.75 ± 0.89	4.15 ± 0.66 *
n-6 PUFA	27.33 ± 2.29	30.48 ± 2.09
18:3n-3 (LNA)	2.72 ± 0.12	2.96 ± 0.24
20:5n-3 (EPA)	7.69 ± 0.66	3.67 ± 0.82 *
22:6n-3 (DHA)	17.92 ± 1.70	12.43 ± 1.87 *
n-3 PUFA	28.33 ± 2.17	19.06 ± 2.78 *
n-3/n-6 PUFA	1.04 ± 0.15	0.62 ± 0.05 *
Nutritional lipid indexes		
AI	0.45 ± 0.02	0.45 ± 0.05
TI	0.27 ± 0.02	0.32 ± 0.02*
hH	2.43 ± 0.11	2.47 ± 0.33
HPI	2.23 ± 0.11	2.25 ± 0.26

Abbreviations: AI, atherogenicity index; TI, thrombogenicity index, h/H, hypocholesterolemic to hypercholesterolemic fatty acids ratio; HPI, health promotion index.

**Table 9:** Comparison of the skin and muscle color features (mean ± SEM) of the largemouth bass (*Micropterus salmoides*) fed trash fish (AF) and an artificial compound diet (ACF) (n = 9 per dietary group; 3 per cage).

	Brightness (L*)		Redness (a*)		Yellowness (b*)	
	TF	ACF	TF	ACF	TF	ACF
Dorsal skin	14.70 ± 0.21	15.14 ± 0.16	0.77 ± 0.36	0.55 ± 0.12	1.25 ± 0.16	1.35 ± 0.09
Lateral skin	29.18 ± 2.86	28.56 ± 1.13	2.44 ± 0.14	2.59 ± 0.12	9.70 ± 0.05	9.87 ± 0.12
Ventral skin	67.56 ± 5.04	68.01 ± 5.24	2.35 ± 0.83	1.85 ± 0.85	11.74 ± 4.22	12.62 ± 2.22
Muscle	36.73 ± 4.57	36.82 ± 4.79	2.68 ± 0.27	2.48 ± 0.16	2.24 ± 0.13	2.53 ± 0.38

**Table 10:** Comparison of muscle texture features (mean ± SEM) of largemouth bass (*Micropterus salmoides*) fed trash fish (AF) and an artificial compound diet (ACF) (n = 9 per dietary group; 3 per cage). The asterisk denotes statistically significant differences between both groups (*t*-test, *p* < 0.05).

Parameter	TF	ACF
Hardness (N)	19.62 ± 0.24	18.00 ± 0.52
Adhesivenes (N.m)	0.21 ± 0.01	0.19 ± 0.01
Cohesiveness	0.15 ± 0.01	0.16 ± 0.03
Springiness (mm)	1.50 ± 0.04	1.40 ± 0.01
Gumminess (N)	3.36 ± 0.28	2.38 ± 0.00 *
Chewiness (N)	5.67 ± 0.04	3.54 ± 0.38 *
Shear force (N)	7.12 ± 0.89	5.95 ± 0.22
Water-holding capacity (%)	7.49 ± 0.17	6.86 ± 0.11
pH	6.43 ± 0.10	6.43 ± 0.06

## Data Availability

Data are available upon request to the corresponding author.
